# Toll-like Receptor Gene Polymorphisms as Predictive Biomarkers for Response to Infliximab in Japanese Patients with Crohn’s Disease

**DOI:** 10.3390/diagnostics15080971

**Published:** 2025-04-10

**Authors:** Jingjing Wei, Hiroki Kurumi, Hajime Isomoto, Ryohei Ogihara, Kayoko Matsushima, Haruhisa Machida, Tetsuya Ishida, Tatsuro Hirayama, Naoyuki Yamaguchi, Yukina Yoshida, Kazuhiro Tsukamoto

**Affiliations:** 1 Department of Endoscopy, The First Affiliated Hospital of Fujian Medical University, Fuzhou 350004, China; weijingjing0208@163.com; 2 Department of Endoscopy, National Regional Medical Center, Binhai Campus of the First Affiliated Hospital of Fujian Medical University, Fuzhou 350212, China; 3 Division of Gastroenterology and Nephrology, Department of Multidisciplinary Internal Medicine, School of Medicine, Faculty of Medicine, Tottori University, Yonago 683-8504, Japan; kurumi_1022_1107@yahoo.co.jp (H.K.); isomoto@tottori-u.ac.jp (H.I.); ryoheiogihara@gmail.com (R.O.); n.ykn.xx22@gmail.com (Y.Y.); 4 Department of Gastroenterology and Hepatology, Nagasaki University Hospital, 1-7-1 Sakamoto, Nagasaki 852-8501, Japan; matsu7281978@yahoo.co.jp (K.M.); haru.machida@gmail.com (H.M.); 5 Department of Gastroenterology, Oita Red Cross Hospital, 3-2-27 Chiyo-machi, Oita 870-0033, Japan; tetsi@oita-u.ac.jp; 6 Department of Pharmacotherapeutics, Nagasaki University Graduate School of Biomedical Sciences, 1-7-1 Sakamoto, Nagasaki 852-8501, Japan; tatsuro_h_20@nagasaki-u.ac.jp; 7 Department of Endoscopy, Nagasaki University Hospital, 1-7-1 Sakamoto, Nagasaki 852-8501, Japan; naoyuki3334@hotmail.com

**Keywords:** Crohn’s disease, infliximab, Toll-like receptors, gene polymorphisms, therapeutic response

## Abstract

**Objectives**: To explore the possible relationship between Toll-like receptor (TLR) gene encoding and a predictive outcome for the loss of response (LOR) to IFX treatment among Japanese patients with Crohn’s disease (CD). **Methods**: An association analysis that involved 25 single-nucleotide polymorphisms (SNPs) across the TLR1, TLR2, TLR4, TLR6, TLR9, and TLR10 genes was performed on a cohort of 127 Japanese patients with CD. The therapeutic responses were evaluated at 10 weeks, 1 year, and 2 years using three different inheritance models. **Results**: The CD patients with a G/G genotype of rs5743565 in TLR1 were significantly less likely in the responders at 10 weeks compared with the non-responders (*p* = 0.023, OR = 0.206). The frequencies of the C/T or T/T genotypes of rs5743604 in the TLR1, G/A, or A/A genotypes of rs13105517 in TLR2, both in the minor allele dominant model, were significantly higher in the responders at 10 weeks as compared with those in the non-responders (*p* = 0.035, OR = 4.401; *p* = 0.017, OR = 5.473). The patients with an A/A genotype of rs13105517 in TLR2 in the minor allele recessive model were significantly less likely in the responders at one year of IFX treatment compared with those in the non-responders (*p* = 0.004, OR = 0.195). **Conclusions**: The polymorphisms of TLR1 and TLR2 can be useful as biomarkers for predicting initial and secondary LOR to IFX in Japanese CD patients. The IFX response in genetic testing may target molecules for new drugs to overcome the non-response and LOR to IFX.

## 1. Introduction

Infliximab (IFX) was the first monoclonal antibody approved for clinical use in treating moderate-to-severe Crohn’s disease (CD) patients who showed an inadequate response to conventional therapy by the FDA in 1998 and was approved in Japan in 2001 [1]. It represented a biopharmaceutical agent characterized as a chimeric immunoglobulin G1 (IgG1) monoclonal antibody that targets tumor necrosis factor-alpha (TNF-α) by binding to it and blocking its interaction with cell receptors [2]. In CD patients, approximately 10~30% exhibit primary non-response (PNR) to IFX therapy, while 23~46% develop a secondary loss of response (LOR) with continued therapy [3]. However, the mechanisms of IFX non-response or secondary LOR remains unclear. Some multifactorial causes are supported by published data, including complex inflammatory signaling, antibodies produced against IFX [4], and abnormal IFX pharmacokinetics [5]. Further investigation is critically needed to uncover the mechanisms of IFX non-response and develop targeted interventions to improve the clinical outcomes for more CD patients.

1. Th17/IL-17-signaling-mediated immune response in the pathogenesis of CD

The pathogenesis of inflammatory bowel disease (IBD) involves a dysregulated immune response to alterations in intestinal commensal homeostasis, involving interactions between the gut bacteria [6], immune response [7], genetic susceptibility [8], and environmental triggers [9]. During the past 30 years, immune pathways mediated by Th1 and Th2 cells were considered as key roles in CD and UC, respectively [10]. With the exploration of more and more Th cell subtypes, especially Th17, Th9, and Treg cells [11], the theoretical system has been more extensively elucidated in recent years. Physiologic mucosal Th17 cells contribute to the epithelial barrier function via neutrophil/macrophage recruitment and antimicrobial peptide induction, maintaining an intestinal mucosal defense [12]. Upon the onset of inflammation, dendritic cells can be stimulated by microbial antigens to secrete pro-inflammatory mediators, such as IL-6 and IL-1β, which subsequently induce the pathogenic differentiation of Th17 with the increasing levels of Th17-related cytokines (IL-17, IL-21, and IL-22) [13]. The accumulation of Th17 cells and their associated cytokines (IL-17, IL-21, and IL-22) was observed in the inflammatory lesions of active IBD patients, higher levels of IL-17A and IL-17A mRNA were found in the serum and intestinal tissue of IBD patients when compared with healthy controls [14], and IL-17A could recruit a variety of immune cells by activating the NF-κB and MAPK pathways [15]. These findings imply that the IL-23/IL-17 axis plays key roles in active disease pathogenesis and mucosal injury. Besides inflammatory injury, another study focused on the effect of the Th17/IL-17 axis on intestinal fibrosis, demonstrating the fibrotic progression by activating myofibroblasts and enhancing collagen production via TGF-β, growth factors and novel mediators (TL1A/DR3, Ang-II) [16]. Our previous data also identified the IFX-treatment-response-associated SNPs of IL-17F as promising biomarkers for predicting the therapeutic response to IFX [17].

2. Toll-like receptor (TLR) signaling shapes the Th17/IL-17 axis responses in CD

Other important drivers of Th17 cell proliferation are TLRs signaling pathways, which can promote naive T-cell differentiation into Th17 cells and IL-17 secretion. TLRs are innate immune sensors that recognize pathogen-associated (PAMPs) and damage-associated (DAMPs) molecular patterns, triggering responses to both infectious and non-infectious stimuli [18,19]. In humans, TLR1-10 is expressed in intestinal epithelial cells and leukocytes and can be activated by commensal microbes [20,21,22]. For instance, TLR4 specifically binds lipopolysaccharides [23], while TLR2 recognizes Gram-positive bacterial components (e.g., lipoteichoic acid) and diverse glycolipids/lipoproteins [24]. TLR9 recognizes unmethylated CpG DNA from microbes within endosomes [25], unlike membrane-bound TLRs (TLR1/2/4/6/10) [26]. TLR4 acts as a homodimer, while TLR2 forms heterodimers with TLR1/6/10 [27,28,29,30]. While normally detecting microbial PAMPs, TLRs initiate NF-κB-mediated cytokine production to combat pathogens [31]. TLR gene polymorphisms are related with an elevated susceptibility to CD. TLR1/2/6/9 polymorphisms may be involved in disease progression in IBD patients [32,33]. However, further genetic evidence shows that the Saudi population with three major CARD15/NOD2 variant alleles and the CD14-159C/T polymorphism but not TLR4 (Thr399Il) are more susceptible to CD [34].

TLRs play a crucial role in infection defense, but overactivation may lead to inflammatory conditions and autoimmune disorders across acute and chronic spectrums [35]. Inappropriate TLR activation could lead to an imbalance in intestinal homeostasis, thereby resulting in a primary non-response and subsequent LOR to IFX therapy in CD patients. Genetic evidence suggests that TLRs are strongly associated with both a CD pathology and LOR to IFX treatment. In a Danish patient study, polymorphisms in TLR1/2/4/6/9/10 were associated with an anti-TNF treatment response [36]. Given the role of TLRs in inflammation, TLRs inhibitors are being developed as potential therapeutic drugs for inflammatory and autoimmune disorders [37,38].

This study aimed to explore TLR gene polymorphisms as predictive indicators of IFX treatment in Japanese CD patients. Based on the evidence, the IFX-treatment-related financial burden and side effects, such as increased susceptibility to infections, autoimmune disorders, and malignancies, could be prevented in non-responders to IFX. This will be meaningful in clinical decision-making by predicting IFX responsiveness in CD patients before therapy.

## 2. Materials and Methods

A multicenter cohort of 127 Japanese CD patients that initiated IFX therapy between 2004 and 2012 was prospectively evaluated across three tertiary care institutions: Oita Red Cross Hospital, Nagasaki Harbor Medical Center City Hospital, and Nagasaki University Hospital. This study was approved by institutional ethics committees (approval ID: 110926210) in compliance with the Declaration of Helsinki, which required written informed consent from all participants prior to enrollment.

**Figure 1:**
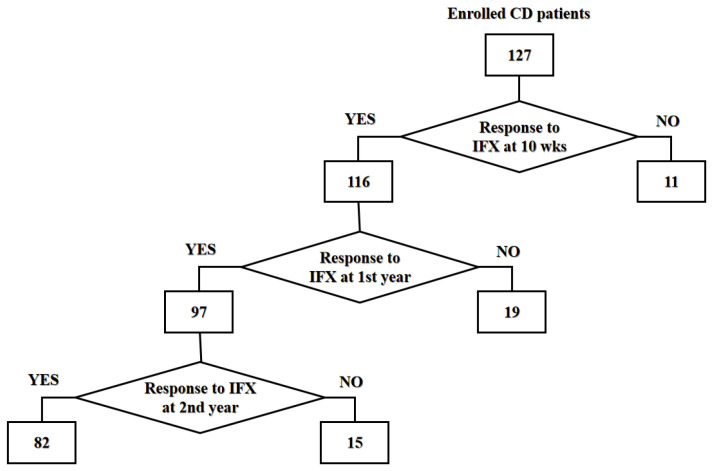
Flowchart of study design.

The treatment efficacy was stratified using a composite clinical endpoint: the responders demonstrated sustained Crohn’s Disease Activity Index (CDAI) scores <150 alongside concurrent improvements in symptomatology, biochemical markers, or endoscopic findings across the treatment intervals, whereas the non-responders exhibited static/elevated CDAI values (>150, indicative of active disease) or clinical deterioration. Longitudinal monitoring comprised sequential evaluations at 10 weeks post-induction (*n* = 127), followed by annual assessments of the initial responders (*n* = 116, 91.3%) over a two-year period. Secondary LOR occurred in 19/116 (16.3%) and 15/97 (15.5%) patients at years 1 and 2, respectively, with demographic and baseline clinical parameters detailed in Table 1 (study schema: Figure 1).

**Figure 2:**
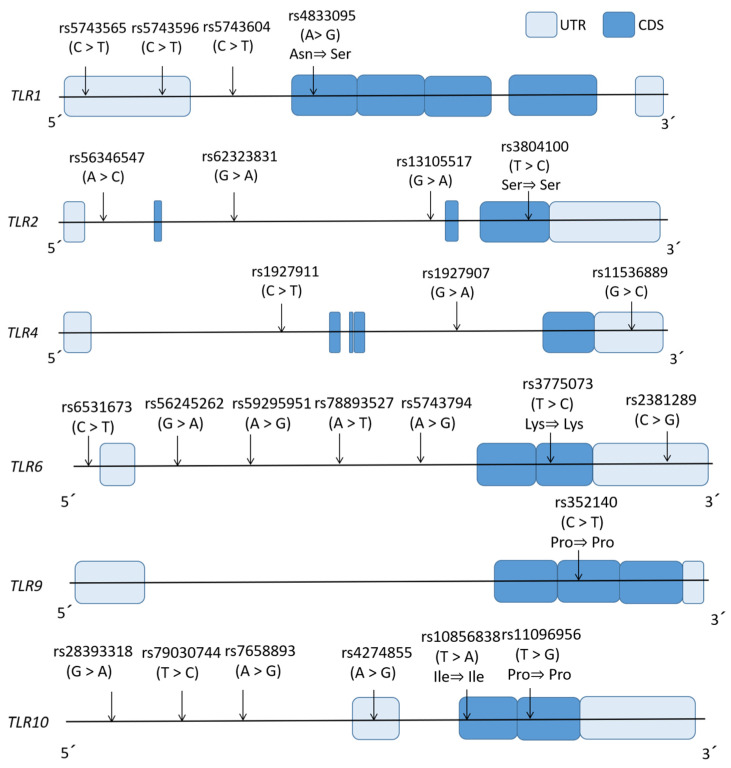
Locations of the genotyped tag SNP sites in TLR1/2/4/6/9/10. Horizontal lines show the genomic sequence of each gene. The light and dark blue boxes indicate the positions of the untranslated regions and coding sequence, respectively. Arrows indicate the locations of all genotyped tag SNP sites in this study, with their names and major/minor alleles presented above. Several tag SNPs were located within exons, with or without residue changes.

The genetic analysis focused on six TLR loci (TLR1, TLR2, TLR4, TLR6, TLR9, TLR10), with tag SNPs selected from the 1000 Genomes Project Phase 3 Tokyo Japanese cohort (hg19/GRCh37) using Haploview v4.2 (MAF ≥ 0.2, LD r^2^ ≥ 0.8) [39,40]. The gene architectures illustrating untranslated regions, coding sequences, and SNP positions (including exonic variants with/without amino acid substitutions) are depicted in Figure 2. Peripheral blood-derived genomic DNA isolated via the WB-Rapid Kit (Fujifilm Wako Chemicals. Co., Osaka, Japan) served as a template for the tripartite genotyping of 25 SNPs.

The PCR-RFLP analyses employed GeneAmp 9700/T100 thermal cyclers (Thermo Fisher Scientific. Co., Waltham, MA, USA) with a GoTaq Green Master Mix (Promega. Co., Tokyo, Japan), followed by restriction enzyme digestion (Table 2) and electrophoretic separation on polyacrylamide/agarose gels visualized via ethidium bromide staining. Sanger sequencing utilized ExoSAP-IT-purified amplicons sequenced with BigDye Terminator v3.1 on ABI 3100/3130xl platforms (Thermo Fisher Scientific. Co., Waltham, MA, USA), while high-resolution melting (HRM) profiling was conducted on a LightCycler 480 system using SYTO9/DMSO-enhanced reactions (Invitrogen Life Technologies, Carlsbad, CA, USA) analyzed through derivative curve modeling (Gene-Scanning v1.3) (Roche Diagnostic. Co., Tokyo, Japan).

The statistical workflows integrated dual-platform validation (SPSS v20/Prism 6), beginning with baseline comparisons via Mann–Whitney U and Fisher’s exact tests. Genetic associations were evaluated under allelic/dominant/recessive models using SNPAlyze v7.0 for Hardy–Weinberg equilibrium and linkage disequilibrium analyses, with multivariate logistic regression modeling of the gene–treatment interactions. The significance thresholds were set at *p* < 0.05 or 95% confidence intervals, excluding unity.

## 3. Results

### 3.1. Clinical Characteristics of Study Population

No significant differences were observed between the responders and non-responders groups (Table 1). Finally, a total number of 28 CD patients (25/127, 19.7%) underwent surgeries during the therapy. The ratio of surgeries in the non-responders of IFX-treated CD patients was 54.5% (6/11) at 10 wks, 47.4% (9/19) at year 1, and 66.7% (10/15) at year 2.

### 3.2. Polymorphisms Associated with the Response to IFX at the 10-Week Treatment

Statistical analyses showed that the patients with a G/G genotype of rs5743565 in TLR1 in the responders in the minor allele recessive model were significantly less likely to respond to IFX at the 10-week treatment as compared with the non-responders (*p* = 0.023, OR = 0.206; Table 3), suggesting a 4.9-fold higher chance of non-response to IFX. Conversely, the patients with an A/A or A/G genotype of rs5743565 had a 4.9-fold higher likelihood of responding to IFX.

Meanwhile, the frequency of the C/T or T/T genotype of rs5743604 in TLR1 in the minor allele dominant model was significantly higher in the responders at the 10-week treatment as compared with that in the non-responders (*p* = 0.035, OR = 4.401; Table 3), indicating a 4.4-fold increase in the response to IFX. In contrast, a C/C genotype of rs5743604 was associated with a 4.4-fold decrease in the response to IFX.

In addition, the patients with a G/A or A/A genotype of rs13105517 in TLR2 in the minor allele dominant model were significantly increased in the responders at the 10-week treatment in comparison with those in the non-responders (*p* = 0.017, OR = 5.473; Table 3). This indicated a 5.5-fold higher likelihood of responding to IFX. Conversely, the patients with a G/G genotype of rs13105517 were 5.5 times more likely to be non-responders to IFX.

### 3.3. Interaction of the Genetic Factors in Response to IFX at the 10-Week Treatment

The differences in the genotype frequencies between the responders and non-responders revealed several genetic factors associated with the IFX response at the 10-week treatment: the A/A or A/G genotype of rs5743565 in TLR1, the C/T or T/T genotype of rs5743604 in TLR1, and the G/A or A/A genotype of rs13105517 in TLR2. Further multivariate logistic regression analysis revealed that the A/A or A/G genotype of rs5743565 in TLR1 and the G/A or A/A genotype of rs13105517 in TLR2 independently contributed to the IFX response (*p* = 0.015 and 0.014, respectively; Table 4).

### 3.4. Verification of Genetic Test to Predict the Response to IFX at the 10-Week Treatment

To better predict the IFX response in the CD patients at the 10-week treatment, a genetic test was conducted using either a single genetic factor (TLR1 or TLR2) or a combination of these independent genetic factors as biomarkers. The combination of the A/A or A/G genotype of rs5743565 in TLR1 and the G/A or A/A genotype of rs13105517 in TLR2 showed a significant response to IFX (*p* = 0.024, OR = 5.735; Table 5). For this genetic test, the sensitivity, specificity, positive predictive value (PPV), and negative predictive value (NPV) were estimated at 56.0%, 81.8%, 97.0%, and 15.0%, respectively. This combination of biomarkers showed a better specificity and PPV.

### 3.5. Polymorphisms Associated with the IFX Response at the 1-Year Treatment

The statistical analyses showed that the patients with an A/A genotype of rs13105517 in TLR2 in the responders in the minor allele recessive model were significantly less likely to respond to the IFX at the 1-year treatment than those in the non-responders (*p* = 0.004, OR = 0.195; Table 6), indicating a 5.1-fold decrease in the IFX response. In contrast, the patients with a G/G or G/A genotype of rs13105517 showed a 5.1-fold higher response to IFX.

### 3.6. Verification of Genetic Testing for Predicting the IFX Response at the 1-Year Treatment

To better predict the IFX response in CD patients at the 1-year treatment, a genetic test with the G/G or G/A genotype of rs13105517 was conducted. The sensitivity, specificity, PPV, and NPV of this test were 91.8%, 31.6%, 87.3%, and 42.9%, respectively (Table 7). This biomarker demonstrated a superior sensitivity and PPV.

### 3.7. Polymorphisms Associated with the IFX Response at the 2-Year Treatment

There were no significant differences in the allele and genotype frequencies of tag SNPs between the responders and non-responders at the 2-year treatment (Table 8).

## 4. Discussion

**Figure 3:**
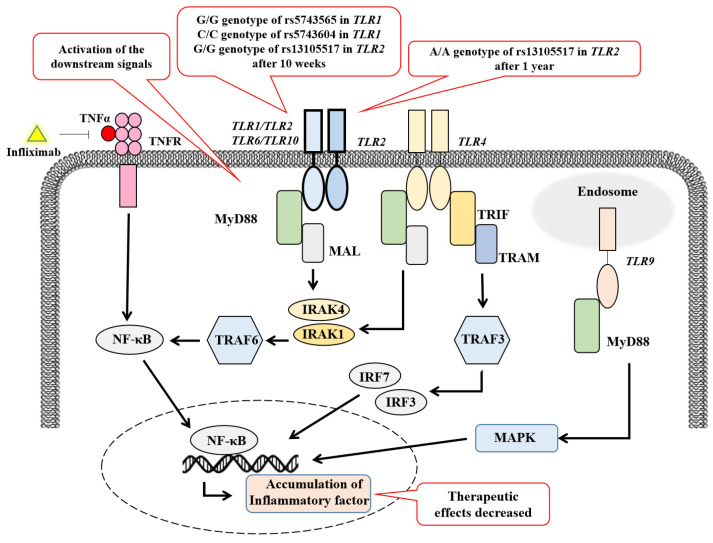
The putative mechanism of non-response and LOR to IFX.

Our results suggest that TLR1 and TLR2 may be involved in both the initial non-response and SLOR to IFX in Japanese CD patients. However, the functional roles of rs5743565 and rs5743604 in TLR1 and rs13105517 in TLR2 remain unclear. The HaploReg analysis indicated that rs5743565 and its highly related variants with high LD (r^2^ ≥ 0.8) could alter the transcription or chromatin states. This in silico analysis showed different affinities to more than 20 transcription factors for the reference and alternative alleles of rs5743565 and several variants with a high LD. In contrast, since rs5743604 does not have a high LD with other common variants, it demonstrated different affinities for the four transcription factors and distinct expression quantitative trait loci between its reference and alternative alleles. rs13105517 and variants with high LDs can also predict different affinities for several transcription factors [41]. Based on these results, the TLR1 and TLR2 polymorphisms could induce the gain of function in CD patients with specific genotypes: the G/G genotype of rs5743565 in TLR1, the C/C genotype of rs5743604 in TLR1 at the 10-week treatment, and the A/A genotype of rs13105517 in TLR2 at both the 10-week and 1-year treatments. These changes could contribute to the activation of intracellular downstream signals in the TLR signaling pathway, which eventually resulted in the acceleration of chronic intestinal inflammatory processes. As shown in Figure 3, persistent intestinal inflammation could diminish the IFX efficacy, which led to both an initial non-response to IFX at the 10-week treatment and a secondary LOR to IFX at the 1-year treatment. Some evidence shows that a transcriptional dysregulation of circulating monocytes would induce hyperactivation of the pro-inflammatory pathways, contributing to the resistance to IFX treatment [42]. Another activated pro-inflammatory pathway mediated by IL-1β, IL-6, and IFN-γ in CD patients, different from TNF-α signaling, might be resistant to anti-TNF treatment [36], suggesting that treatment strategies should be broadened for CD patients.

Conversely, the CD patients with specific genotypes could experience the loss of function of TLR1 and TLR2. This included patients with the A/A or G/A genotype of rs5743565 in TLR1, C/T or T/T genotype of rs5743604 in TLR1 at the 10-week treatment, and G/A or G/G genotype of rs13105517 in TLR2 at both the 10-week and 1-year treatments. This could inhibit the intracellular downstream signals, which eventually resulted in the continued suppression of signals in the TNFR signaling pathway. Therefore, these patients could benefit from IFX at the 10-week and 1-year treatments in this study.

Gut homeostasis involved in the mechanism of IFX therapy is under investigation. A functional I602S SNP in TLR1 can regulate the innate immune response to lipopeptides caused by a pathogen infection, which could potentially affect inflammatory pathways [43], although there is no study about this allele in the Japanese population [44]. An in silico analysis of microarray experiment GSE16879 in the GEO database revealed higher expressions of TLR1 and TLR2 in CD patients resistant to IFX treatment. After an IFX treatment, the downregulation of TLR2 was found in patients who responded to IFX, but remained unchanged in non-responders [45] (data accessible at NCBI GEO database [46], accession GSE16879). Evidence suggests that the polymorphisms of TLR1 and TLR2 might be strongly related to the non-response and LOR to IFX in CD patients.

In our study, the combination of the G/A or A/A genotype of rs5743565 in TLR1 and the G/A or A/A genotype of rs13105517 in TLR2 proved to be a useful biomarker for predicting the IFX response, showing a high OR of 5.735, specificity of 81.8%, and PPV of 97.0% in genetic testing at the 10-week treatment. A higher PPV indicates that the CD patients with this combination polymorphism were more likely to respond successfully to the IFX treatment at the 10-week mark. Likewise, the G/G or G/A genotype of rs13105517 in TLR2 could be considered a useful biomarker for predicting an IFX response, with a higher OR of 3.736, sensitivity of 91.8%, and PPV of 87.3% in genetic testing at the 1-year mark after treatment, indicating that the CD patients with this polymorphism are more likely to respond successfully to IFX treatment and be continuously effective for at least 1 year.

Taken together, our findings demonstrate significant associations between TLR1/2 genetic variants and a differential response to IFX in CD patients. The results should be interpreted with consideration of its limitations. First, the data were based on a small cohort limited to the Japanese population; future studies should be considered to investigate diverse ethnic groups. The optimal combination of genetic biomarkers for a better prediction of IFX effectiveness should also be discussed in the future. Furthermore, the exact mechanisms are essential for the refinement of therapeutic strategies for IFX non-response in CD patients.

## Figures and Tables

**Table 1:** Comparison of the characteristics between responders and non-responders to IFX for the CD patients.

Characteristics	Groups		*p*-Value
	Responders	Non-Responders	
10 weeks (n = 127)			
Number (%)	116 (91.3)	11 (8.7)	N/A
Age, mean ± SD (years)	35.1 ± 12.3	35.2 ± 7.1	0.662
Male/female (%)	67/49 (57.8/42.2)	10/1 (90.9/9.1)	0.049
1 year (*n* = 116)			
Number (%)	97 (83.6)	19 (16.4)	N/A
Age, mean ± SD (years)	34.7 ± 12.8	37.3 ± 9.0	0.234
Male/female (%)	56/41 (57.7/42.3)	11/8 (57.9/42.1)	0.990
2 years (*n* = 97)			
Number (%)	82 (84.5)	15 (15.5)	N/A
Age, mean ± SD (years)	35.0 ± 13.3	32.8 ± 9.5	0.787
Male/female (%)	48/34 (58.5/41.5)	8/7 (53.3/46.7)	0.708

Abbreviations: IFX, infliximab; CD, Crohn’s disease; SD, standard deviation; N/A, not applicable.

**Table 2:** Information on the genotyping of Tag SNPs in the candidate genes.

Gene	Tag SNP	Major>Minor	dbSNP Function	Locus	Sequence of Primer (5’ to 3’)		Annealing Temperature (°C)	Cycle Number	Analytic Method (Restriction Enzyme)
					Forward	Reverse			
*TLR1*	rs5743565	A>G	5’UTR	4p14	CCTCTGGGAATAACACCTCGT	GAAGGCCCCAGAGAGAAAAA	58	30	PCR-RFLP (*Hin*F I)
	rs5743596	C>T	5’UTR		AAATTTCCGGGTCTTTCAGC	ATCTGGGTTTTGGAGCCTTC	55	50	HRM with non-labeled probe
	rs5743604	C>T	Intronic		GAGCAGTCCCAATACCACCA	AGGAGAAGGCCTTGTGACAGT	55	30	PCR-RFLP (*Bmg*B I)
	rs4833095	C>T	Missense		AAACCAGCTGGAGGATCCTAAT	CATTGTGTTCCCCACAAACA	57	50	HRM with non-labeled probe
*TLR2*	rs56346547	A>C	Intronic		GCGGACTTTCCCTTTTGCTT	GCTTCGCTGGTGTCCACATT	57	30	PCR-RFLP (*Bsm*F I)
	rs62323831	A>G	Intronic	4q31.3	GGCCAAATCTGGGGCTAGTT	CATATGCGAATCCCGACTCC	58	50	HRM with non-labeled probe
	rs13105517	A>G	Intronic	3p21.2	ACTTCCTGGATTGCGGTGAT	AAACTCGAGGCAGACCAAGG	60	30	PCR-RFLP (Bts I)
	rs3804100	C>T	Synonymous		TGCCTGGCCCTCTCTACAAA	GAGTTGCGGCAAATTCAAAG	55	30	PCR-RFLP (*Hpy*CH4III)
*TLR4*	rs1927911	C>T	Intronic	9q33.1	TCATGGCCCAGATTTTGACA	AGCTGGCTTCTGCAAGGAAT	55	30	PCR-RFLP (*Eco*130 I)
	rs1927907	A>G	Intronic		GGCTGCCTGGTCTATCACAA	TTCAACCCTTGCTGCTTTCTC	56	30	PCR-RFLP (*Hph* I)
	rs11536889	C>G	3’UTR		TTTGGGCTAGAGGCAGGAAG	TTTCCATTCCCTCCAGCAGT	60	30	PCR-RFLP (*Hpy*CH4III)
*TLR6*	rs6531673	C>T	Intronic	4p14	GCATGATACTGCAGAAAGCAAGTACC	GCAACTCAAGTCATCCAGTGAA	59	50	HRM with non-labeled probe
	rs56245262	A>T	Intronic		ATCCACTTGGCCACTGAAAA	GGAGAATGGAGTGTGGCAGT	56	30	PCR-RFLP (*Bcu* I)
	rs59295951	A>G	Intronic		TCTTTTATCCTTCCCCACCA	GGTCACTGGTTGCAGCAGAT	53	50	HRM with non-labeled probe
	rs78893527	C>G	Intronic		CAACCCTGAATCTCCACACC	CTCTGGGGAATGCACACTTT	53	50	HRM with non-labeled probe
	rs5743794	G>A	Intronic		ACTGAGTTGCCTTTGCTCGT	GCCAGATAACTGACACCACCT	55	50	HRM with non-labeled probe
	rs3775073	A>G	Synonymous		GCTGGAATTCTTTGGAATCTGG	GCAAAGCTTCCAGTTTTACGAC	55	50	HRM with non-labeled probe
	rs2381289	T>C	3’UTR		AGGAAGGCCAAGCAGATTTT	AGCGAGGGCTTCATTTTTCT	53	50	HRM with non-labeled probe
*TLR9*	rs352140	C>T	Synonymous		TTCCAGTTTGGGCAGGAAGT	GTCACGGAACAACCTGGTGA	58	30	PCR-RFLP (*Bsh*1236 I)
*TLR10*	rs28393318	G>A	Intronic	4p14	TCCATCAGATCTGCCCCTAC	TGAGAGCGTGGGTTTCTTTT	55	50	HRM with non-labeled probe
	rs79030744	T>C	Intronic		AGGAGCTAAAGCCCAGAGGT	TCGGTCCTTAGGATGTCGTT	55	50	HRM with non-labeled probe
	rs7658893	A>G	Intronic		TGGTGGCAGTTATCAGGTCA	AACTGCCAGGGTCCTATCAA	55	50	HRM with non-labeled probe
	rs4274855	A>G	5’UTR		CAAAGGCTCACAATGTCTGG	TCAGAGCATTGGCTGAGAAG	57	50	HRM with non-labeled probe
	rs10856838	T>A	Synonymous		GGGTTTTGAGCTCATCTTCATC	TTGGAGCAGTTGGTCATCAG	55	50	HRM with non-labeled probe
	rs11096956	T>G	Synonymous		CACAAATGCCACACATGCTT	TCAGATCCAAGTGTTCCAAGG	52	50	HRM with non-labeled probe

Abbreviations: SNP, single nucleotide polymorphism; 3’-UTR, 3’-untranslated region; PCR, polymerase chain reaction; RFLP, restriction fragment length polymorphism; HRM, high-resolution melting.

**Table 3:** Allele and genotype comparisons in three inheritance models between responders and non-responders to IFX at the 10-weeks treatment for the CD patients.

Gene	Tag SNP (Major>Minor)	Genotype	Groups		Inheritance Model *	Genotype Comparison			Gene	Tag SNP (Major>Minor)	Genotype	Groups		Inheritance Model *	Genotype Comparison		
			Responders	Non-Responders		*p*-Value	OR	95% CI				Responders	Non-Responders		*p*-Value	OR	95% CI
			(*n*, %)	(*n*, %)								(*n*, %)	(*n*, %)				
*TLR1*	rs5743565	MAF	0.405	0.636	Allele	0.036	0.389	0.157–0.964	*TLR6*	rs6531673	MAF	0.315	0.318	Allele	0.973	0.984	0.385–2.516
	A>G	A/A	39 (33.6)	2 (18.2)						C>T	C/C	52 (44.8)	4 (36.4)				
		A/G	60 (51.7)	4 (36.4)	Dominant	0.501	0.439	0.090–2.130			C/T	55 (47.4)	7 (63.6)	Dominant	0.754	0.703	0.195–2.534
		G/G	17 (14.7)	5 (45.5)	Recessive	0.023	0.206	0.057–0.751			T/T	9 (7.8)	0 (N/A)	Recessive	1.000	0.492	0.027–9.021
	rs5743596	MAF	0.328	0.455	Allele	0.229	0.585	0.242–1.413		rs56245262	MAF	0.263	0.318	Allele	0.448	1.434	0.563–3.651
	C>T	C/C	51 (44.0)	4 (36.4)						A>T	A/A	39 (33.6)	5 (45.5)				
		C/T	54 (46.6)	4 (36.4)	Dominant	0.756	0.728	0.202–2.625			A/T	61 (52.6)	5 (45.5)	Dominant	0.512	1.645	0.472–5.731
		T/T	11 (9.5)	3 (27.3)	Recessive	0.104	0.279	0.065–1.209			T/T	16 (13.8)	1 (9.1)	Recessive	1.000	1.600	0.192–13.369
	rs5743604	MAF	0.457	0.273	Allele	0.096	2.243	0.848–5.938		rs59295951	MAF	0.280	0.318	Allele	0.705	0.834	0.325–2.139
	C>T	C/C	33 (28.4)	7 (63.6)						A>G	A/A	58 (50.0)	5 (45.5)				
		C/T	60 (51.7)	2 (18.2)	Dominant	0.035	4.401	1.208–16.026			A/G	51 (44.0)	5 (45.5)	Dominant	1.000	0.833	0.241–2.884
		T/T	23 (19.8)	2 (18.2)	Recessive	1.000	1.113	0.225–5.507			G/G	7 (6.0)	1 (9.1)	Recessive	0.526	0.642	0.072–5.757
	rs4833095	MAF	0.233	0.318	Allele	0.471	1.408	0.553–3.587		rs78893527	MAF	0.164	0.182	Allele	0.768	0.881	0.283–2.750
	C>T	C/C	43 (37.1)	6 (54.5)						C>G	C/C	81 (69.8)	7 (63.6)				
		C/T	54 (46.6)	3 (27.3)	Dominant	0.334	2.037	0.586–7.077			C/G	32 (27.6)	4 (36.4)	Dominant	0.736	0.756	0.208–2.750
		T/T	19 (16.4)	2 (18.2)	Recessive	1.000	0.881	0.176–4.405			G/G	3 (2.6)	0 (N/A)	Recessive	1.000	1.410	0.068–29.050
*TLR2*	rs56346547	MAF	0.246	0.273	Allele	0.779	0.869	0.324–2.325		rs5743794	MAF	0.371	0.409	Allele	0.722	0.851	0.349–2.073
	A>C	A/A	63 (54.3)	6 (54.5)						G>A	G/G	45 (38.8)	4 (36.4)				
		A/C	49 (42.2)	4 (36.4)	Dominant	1.000	1.009	0.292–3.495			G/A	56 (48.3)	5 (45.5)	Dominant	1.000	0.902	0.250–3.255
		C/C	4 (3.4)	1 (9.1)	Recessive	0.369	0.357	0.036–3.509			A/A	15 (12.9)	2 (18.2)	Recessive	0.642	0.668	0.132–3.396
	rs62323831	MAF	0.319	0.545	Allele	0.032	0.390	0.161–0.944		rs3775073	MAF	0.332	0.318	Allele	0.896	0.939	0.368–2.400
	G>A	G/G	50 (43.1)	2 (18.2)						A>G	A/A	51 (44.0)	5 (45.5)				
		G/A	58 (50.0)	6 (54.5)	Dominant	0.198	0.293	0.061–1.418			A/G	53 (45.7)	5 (45.5)	Dominant	1.000	1.062	0.307–3.678
		A/A	8 (6.9)	3 (27.3)	Recessive	0.055	0.198	0.044–0.893			G/G	12 (10.3)	1 (9.1)	Recessive	1.000	1.154	0.136–9.814
	rs13105517	MAF	0.397	0.136	Allele	0.020	4.161	1.197–14.472		rs2381289	MAF	0.435	0.455	Allele	0.862	0.925	0.384–2.227
	G>A	G/G	38 (32.8)	8 (72.7)						T>C	T/T	34 (29.3)	3 (27.3)				
		G/A	64 (55.2)	3 (27.3)	Dominant	0.017	5.473	1.374–21.786			T/C	63 (54.3)	6 (54.5)	Dominant	1.000	0.904	0.226–3.617
		A/A	14 (12.1)	0 (N/A)	Recessive	0.609	3.254	0.182–58.250			C/C	19 (16.4)	2 (18.2)	Recessive	1.000	0.881	0.176–4.405
	rs3804100	MAF	0.349	0.500	Allele	0.159	0.536	0.223–1.291	*TLR10*	rs28393318	MAF	0.470	0.591	Allele	0.277	1.630	0.671–3.962
	T>C	T/T	48 (41.4)	3 (27.3)						A>G	A/A	31 (26.7)	2 (18.2)				
		T/C	55 (47.4)	5 (45.5)	Dominant	0.524	0.531	0.134–2.106			A/G	61 (52.6)	5 (45.5)	Dominant	0.727	0.609	0.125–2.977
		C/C	13 (11.2)	3 (27.3)	Recessive	0.144	0.337	0.079–1.430			G/G	24 (20.7)	4 (36.4)	Recessive	0.258	0.457	0.123–1.689
*TLR4*	rs1927911	MAF	0.366	0.227	Allele	0.246	1.966	0.700–5.519		rs79030744	MAF	0.259	0.227	Allele	1.000	1.186	0.419–3.355
	C>T	C/C	51 (44.0)	7 (63.6)						T>C	T/T	63 (54.3)	7 (63.6)				
		C/T	45 (38.8)	3 (27.3)	Dominant	0.343	2.230	0.619–8.039			T/C	46 (39.7)	3 (27.3)	Dominant	0.753	1.472	0.409–5.305
		T/T	20 (17.2)	1 (9.1)	Recessive	0.690	2.083	0.252–17.212			C/C	7 (6.0)	1 (9.1)	Recessive	0.526	0.642	0.072–5.757
	rs1927907	MAF	0.366	0.364	Allele	0.980	1.012	0.408–2.511		rs7658893	MAF	0.483	0.364	Allele	0.285	1.633	0.660–4.042
	G>A	G/G	44 (37.9)	4 (36.4)						A>G	A/A	30 (25.9)	3 (27.3)				
		G/A	59 (50.9)	6 (54.5)	Dominant	1.000	0.935	0.259–3.378			A/G	60 (51.7)	8 (72.7)	Dominant	1.000	1.075	0.268–4.318
		A/A	13 (11.2)	1 (9.1)	Recessive	1.000	1.262	0.149–10.672			G/G	26 (22.4)	0 (N/A)	Recessive	0.119	6.735	0.384–118.200
	rs11536889	MAF	0.310	0.455	Allele	0.167	0.540	0.223–1.307		rs4274855	MAF	0.315	0.318	Allele	0.973	0.984	0.385–2.516
	G>C	G/G	53 (45.7)	2 (18.2)						C>T	C/C	52 (44.8)	4 (36.4)				
		G/C	54 (46.6)	8 (72.7)	Dominant	0.112	0.264	0.055–1.276			C/T	55 (47.4)	7 (63.6)	Dominant	0.754	0.703	0.195–2.534
		C/C	9 (7.8)	1 (9.1)	Recessive	1.000	0.841	0.096–7.331			T/T	9 (7.8)	0 (N/A)	Recessive	1.000	2.033	0.111–37.270
*TLR9*	rs352140	MAF	0.289	0.500	Allele	0.787	0.886	0.370–2.125		rs10856838	MAF	0.159	0.136	Allele	0.774	1.441	0.408–5.089
	C>T	C/C	28 (24.1)	3 (27.3)						T>A	T/T	76 (65.5)	8 (72.7)				
		C/T	67 (57.8)	5 (45.5)	Dominant	0.729	1.179	0.293–4.748			T/A	37 (31.9)	3 (27.3)	Dominant	0.749	1.404	0.353–5.583
		T/T	21 (18.1)	3 (27.3)	Recessive	0.434	0.589	0.144–2.411			A/A	3 (2.6)	0 (N/A)	Recessive	1.000	0.709	0.034–14.610
										rs11096956	MAF	0.371	0.409	Allele	0.722	0.851	0.349–2.073
										G>A	G/G	45 (38.8)	4 (36.4)				
											G/A	56 (48.3)	5 (45.5)	Dominant	1.000	0.902	0.250–3.255
											A/A	15 (12.9)	2 (18.2)	Recessive	0.642	0.668	0.132–3.396

* Allele: allele Model; dominant: the minor allele dominant model; recessive: the minor allele recessive Model. Abbreviations: IFX, infliximab; CD, Crohn’s disease; SNP, single nucleotide polymorphism; OR, odds ratio; CI, confidence interval; MAF, minor allele frequency; N/A, not applicable.

**Table 4:** The interaction of genetic factors for response to IFX at the 10-weeks treatment for the CD patients.

Factor	OR (95% CI)	*p*-Value *
A/A or A/G genotype of rs5743565 in TLR1	5.593 (1.407–22.239)	0.015
G/A or A/A genotype of rs13105517 in TLR2	6.124 (1.454–25.789)	0.014

* Factors were statistically analyzed by multivariate logistic regression analysis. Abbreviations: IFX, infliximab; CD, Crohn’s disease; OR, odds ratio; CI, confidence interval.

**Table 5:** Combination of genetic factors determined by genetic test for response to IFX at the 10-weeks treatment for the CD patients.

Biomarker	*TLR1*	*TLR2*	Statistical Results		Genetic Test			
	rs5743565	rs13105517	OR (95% CI)	*p*-Value *	Sensitivity	Specificity	PPV	NPV
Marker 1	A/A or A/G	–	4.853 (1.331–17.690)	0.023	85.3	45.5	94.3	22.7
Marker 2	–	G/A or A/A	5.473 (1.374–21.790)	0.017	67.2	72.7	96.3	17.4
Marker 3	A/A or A/G	G/A or A/A	5.735 (1.186–27.730)	0.024	56.0	81.8	97.0	15.0

* Factors were statistically analyzed by Fisher’s exact test. Abbreviations: IFX, infliximab; CD, Crohn’s disease; OR, odds ratio; CI, confidence interval; PPV, positive predictive value; NPV, negative predictive value.

**Table 6:** Allele and genotype comparisons in three inheritance models between responders and non-responders to IFX at the 1-year treatment for the CD patients.

Gene	Tag SNP (Major>Minor)	Genotype	Groups		Inheritance Model *	Genotype Comparison			Gene	Tag SNP (Major>Minor)	Genotype	Groups		Inheritance Model *	Genotype Comparison		
			Responders	Non-Responders		*p*-Value	OR	95% CI				Responders	Non-Responders		*p*-Value	OR	95% CI
			(*n*, %)	(*n*, %)								(*n*, %)	(*n*, %)				
*TLR1*	rs5743565	MAF	0.402	0.421	Allele	0.827	0.925	0.457–1.871	*TLR6*	rs6531673	MAF	0.330	0.237	Allele	0.259	1.586	0.709–3.550
	A>G	A/A	33 (34.0)	6 (31.6)						C>T	C/C	41 (42.3)	11 (57.9)				
		A/G	50 (51.5)	10 (52.6)	Dominant	0.837	0.895	0.312–2.570			C/T	48 (49.5)	7 (36.8)	Dominant	0.210	1.878	0.694–5.084
		G/G	14 (14.4)	3 (15.8)	Recessive	1.000	0.900	0.232–3.494			T/T	8 (8.2)	1 (5.3)	Recessive	1.000	1.618	0.190–13.755
	rs5743596	MAF	0.330	0.316	Allele	0.866	1.067	0.506–2.251		rs56245262	MAF	0.418	0.316	Allele	0.242	1.553	0.740–3.259
	C>T	C/C	42 (43.3)	9 (47.4)						A>T	A/A	30 (30.9)	9 (47.4)				
		C/T	46 (47.4)	8 (42.1)	Dominant	0.744	1.179	0.440–3.160			A/T	53 (54.6)	8 (42.1)	Dominant	0.165	2.010	0.741–5.453
		T/T	9 (9.3)	2 (10.5)	Recessive	1.000	0.869	0.172–4.384			T/T	14 (14.4)	2 (10.5)	Recessive	1.000	1.434	0.298–6.897
	rs5743604	MAF	0.479	0.342	Allele	0.120	1.771	0.856–3.663		rs59295951	MAF	0.263	0.368	Allele	0.185	0.611	0.294–1.272
	C>T	C/C	26 (26.8)	7 (36.8)						A>G	A/A	50 (51.5)	8 (42.1)				
		C/T	49 (50.5)	11 (57.9)	Dominant	0.375	1.593	0.566–4.484			A/G	43 (44.3)	8 (42.1)	Dominant	0.452	0.684	0.253–1.847
		T/T	22 (22.7)	1 (5.3)	Recessive	0.116	5.280	0.667–41.841			G/G	4 (4.1)	3 (15.8)	Recessive	0.086	0.229	0.047–1.123
	rs4833095	MAF	0.423	0.263	Allele	0.066	2.050	0.943–4.454		rs78893527	MAF	0.160	0.184	Allele	0.710	0.842	0.340–2.083
	C>T	C/C	33 (34.0)	10 (52.6)						C>G	C/C	69 (71.1)	12 (63.2)				
		C/T	46 (47.4)	8 (42.1)	Dominant	0.125	2.155	0.798–5.821			C/G	25 (25.8)	7 (36.8)	Dominant	0.489	0.696	0.248–1.949
		T/T	18 (18.6)	1 (5.3)	Recessive	0.193	4.102	0.514–32.787			G/G	3 (3.1)	0 (N/A)	Recessive	1.000	0.692	0.034–13.960
*TLR2*	rs56346547	MAF	0.247	0.237	Allele	0.890	1.059	0.468–2.395		rs5743794	MAF	0.381	0.316	Allele	0.444	1.336	0.636–2.808
	A>C	A/A	53 (54.6)	10 (52.6)						G>A	G/G	36 (37.1)	9 (47.4)				
		A/C	40 (41.2)	9 (47.4)	Dominant	0.872	0.922	0.344–2.471			G/A	48 (49.5)	8 (42.1)	Dominant	0.402	1.525	0.566–4.105
		C/C	4 (4.1)	0 (N/A)	Recessive	1.000	1.877	0.097–36.330			A/A	13 (13.4)	2 (10.5)	Recessive	1.000	1.315	0.272–6.369
	rs62323831	MAF	0.335	0.237	Allele	0.235	1.624	0.726–3.632		rs3775073	MAF	0.314	0.421	Allele	0.202	1.586	0.778–3.231
	G>A	G/G	39 (40.2)	11 (57.9)						A>G	A/A	44 (45.4)	7 (36.8)				
		G/A	51 (52.6)	7 (36.8)	Dominant	0.155	2.045	0.754–5.543			A/G	45 (46.4)	8 (42.1)	Dominant	0.494	0.703	0.255–1.938
		A/A	7 (7.2)	1 (5.3)	Recessive	1.000	1.400	0.162–12.092			G/G	8 (8.2)	4 (21.1)	Recessive	0.108	0.337	0.090–1.261
	rs13105517	MAF	0.361	0.579	Allele	0.012	0.411	0.202–0.833		rs2381289	MAF	0.448	0.368	Allele	0.363	1.394	0.680–2.856
	G>A	G/G	35 (36.1)	3 (15.8)						T>C	T/T	27 (27.8)	7 (36.8)				
		G/A	54 (55.7)	10 (52.6)	Dominant	0.111	0.332	0.090–1.220			T/C	53 (54.6)	10 (52.6)	Dominant	0.430	1.512	0.539–4.246
		A/A	8 (8.2)	6 (31.6)	Recessive	0.004	0.195	0.058–0.652			C/C	17 (17.5)	2 (10.5)	Recessive	0.735	1.806	0.381–8.562
	rs3804100	MAF	0.376	0.211	Allele	0.050	2.262	0.984–5.200	*TLR10*	rs28393318	MAF	0.464	0.500	Allele	0.684	1.156	0.576–2.317
	T>C	T/T	37 (38.1)	11 (57.9)						A>G	A/A	28 (28.9)	3 (15.8)				
		T/C	47 (48.5)	8 (42.1)	Dominant	0.110	2.230	0.821–6.053			A/G	48 (49.5)	13 (68.4)	Dominant	0.395	0.462	0.125–1.711
		C/C	13 (13.4)	0 (N/A)	Recessive	0.123	6.231	0.355–109.500			G/G	21 (21.6)	3 (15.8)	Recessive	0.760	1.474	0.392–5.540
*TLR4*	rs1927911	MAF	0.376	0.316	Allele	0.479	1.307	0.622–2.748		rs79030744	MAF	0.278	0.158	Allele	0.121	2.057	0.814–5.198
	C>T	C/C	41 (42.3)	10 (52.6)						T>C	T/T	50 (51.5)	13 (68.4)				
		C/T	39 (40.2)	6 (31.6)	Dominant	0.405	1.518	0.566–4.070			T/C	40 (41.2)	6 (31.6)	Dominant	0.177	2.037	0.715–5.797
		T/T	17 (17.5)	3 (15.8)	Recessive	1.000	1.133	0.297–4.327			C/C	7 (7.2)	0 (N/A)	Recessive	0.597	3.232	0.177–59.030
	rs1927907	MAF	0.392	0.237	Allele	0.070	2.075	0.931–4.625		rs7658893	MAF	0.479	0.500	Allele	0.816	0.921	0.459–1.846
	G>A	G/G	33 (34.0)	11 (57.9)						A>G	A/A	26 (26.8)	4 (21.1)				
		G/A	52 (53.6)	7 (36.8)	Dominant	0.050	2.667	0.978–7.267			A/G	49 (50.5)	11 (57.9)	Dominant	0.777	0.728	0.221–2.396
		A/A	12 (12.4)	1 (5.3)	Recessive	0.691	2.541	0.310–20.790			G/G	22 (22.7)	4 (21.1)	Recessive	1.000	1.100	0.331–3.656
	rs11536889	MAF	0.294	0.395	Allele	0.219	0.638	0.310–1.311		rs4274855	MAF	0.330	0.237	Allele	0.259	1.586	0.709–3.550
	G>C	G/G	47 (48.5)	6 (31.6)						C>T	C/C	41 (42.3)	11 (57.9)				
		G/C	43 (44.3)	11 (57.9)	Dominant	0.177	0.491	0.172–1.398			C/T	48 (49.5)	7 (36.8)	Dominant	0.210	1.878	0.694–5.084
		C/C	7 (7.2)	2 (10.5)	Recessive	0.640	0.661	0.126–3.459			T/T	8 (8.2)	1 (5.3)	Recessive	1.000	1.618	0.190–13.755
*TLR9*	rs352140	MAF	0.454	0.553	Allele	0.263	0.672	0.334–1.352		rs10856838	MAF	0.196	0.132	Allele	0.494	1.608	0.588–4.394
	C>T	C/C	26 (26.8)	2 (10.5)						T>A	T/T	61 (62.9)	15 (78.9)				
		C/T	54 (55.7)	13 (68.4)	Dominant	0.155	0.321	0.069–1.487			T/A	34 (35.1)	3 (15.8)	Dominant	0.201	2.213	0.682–7.184
		T/T	17 (17.5)	4 (21.1)	Recessive	0.747	0.797	0.235–2.701			A/A	2 (2.1)	1 (5.3)	Recessive	0.418	0.379	0.033–4.403
										rs11096956	MAF	0.381	0.316	Allele	0.444	1.336	0.636–2.808
										G>A	G/G	36 (37.1)	9 (47.4)				
											G/A	48 (49.5)	8 (42.1)	Dominant	0.402	1.525	0.566–4.105
											A/A	13 (13.4)	2 (10.5)	Recessive	1.000	1.315	0.272–6.369

* Allele: allele model; dominant: the minor allele dominant model; recessive: the minor allele recessive model. Abbreviations: IFX, infliximab; CD, Crohn’s disease; SNP, single nucleotide polymorphism; OR, odds ratio; CI, confidence interval; MAF, minor allele frequency; N/A, not applicable.

**Table 7:** Genetic factor on genetic test for response to infliximab at the 1-year treatment for the CD patients.

Biomarker	Statistical Results		Genetic Diagnosis			
	OR (95% CI)	*p*-Value *	Sensitivity	Specificity	PPV	NPV
G/G or G/A genotype of rs13105517 in TLR2	3.736 (1.029–13.57)	0.004	91.8	31.6	87.3	42.9

* Factors were statistically analyzed by Fisher’s exact test. Abbreviations: CD, Crohn’s disease; OR, odds ratio; CI, confidence interval; PPV, positive predictive value; NPV, negative predictive value.

**Table 8:** Allele and genotype comparisons in three inheritance models between responders and non-responders to IFX the 2-year treatment for the CD patients.

Gene	Tag SNP (Major>Minor)	Genotype	Groups (*n*,%)		Inheritance Model *	Genotype Comparison			Gene	Tag SNP (Major>Minor)	Genotype	Groups (n,%)		Inheritance Model *	Genotype Comparison			
			Responders (*n*, %)	Non-Responders (*n*, %)		*p*-Value	OR	95% CI				Responders (*n*, %)	Non-Responders (*n*, %)		*p*-Value	OR	95% CI	
*TLR1*	rs5743565	MAF	0.402	0.400	Allele	0.980	1.010	0.456–2.236	*TLR6*	rs6531673	MAF	0.348	0.233	Allele	0.221	1.750	0.708–4.327	
	A>G	A/A	28 (34.1)	5 (33.3)						C>T	C/C	33 (40.2)	8 (53.3)					
		A/G	42 (51.2)	8 (53.3)	Dominant	1.000	0.964	0.300–3.096			C/T	41 (50.0)	7 (46.7)	Dominant	0.345	1.697	0.561–5.131	
		G/G	12 (14.6)	2 (13.3)	Recessive	1.000	1.114	0.223–5.574			T/T	8 (9.8)	0 (N/A)	Recessive	0.351	0.283	0.015–5.164	
	rs5743596	MAF	0.341	0.267	Allele	0.423	1.426	0.597–3.407		rs56245262	MAF	0.433	0.333	Allele	0.309	1.527	0.673–3.465	
	C>T	C/C	35 (42.7)	7 (46.7)						A>T	A/A	25 (30.5)	5 (33.3)					
		C/T	38 (46.3)	8 (53.3)	Dominant	0.775	1.175	0.389–3.547			A/T	43 (52.4)	10 (66.7)	Dominant	1.000	1.140	0.353–3.681	
		T/T	9 (11.0)	0 (N/A)	Recessive	0.347	4.007	0.221–72.59			T/T	14 (17.1)	0 (N/A)	Recessive	0.117	6.562	0.371–116.100	
	rs5743604	MAF	0.470	0.533	Allele	0.520	0.774	0.355–1.689		rs59295951	MAF	0.262	0.267	Allele	0.959	0.977	0.405–2.358	
	C>T	C/C	24 (29.3)	2 (13.3)						A>G	A/A	43 (52.4)	7 (46.7)					
		C/T	39 (47.6)	10 (66.7)	Dominant	0.341	0.372	0.078–1.774			A/G	35 (42.7)	8 (53.3)	Dominant	0.681	0.794	0.263–2.392	
		T/T	19 (23.2)	3 (20.0)	Recessive	1.000	1.206	0.308–4.726			G/G	4 (4.9)	0 (N/A)	Recessive	1.000	1.777	0.091–34.740	
	rs4833095	MAF	0.421	0.433	Allele	0.898	0.950	0.433–2.084		rs78893527	MAF	0.152	0.200	Allele	0.513	0.719	0.267–1.938	
	C>T	C/C	28 (34.1)	5 (33.3)						C>G	C/C	59 (72.0)	10 (66.7)					
		C/T	39 (47.6)	7 (46.7)	Dominant	1.000	0.964	0.300–3.096			C/G	21 (25.6)	4 (26.7)	Dominant	0.759	0.780	0.240–2.529	
		T/T	15 (18.3)	3 (20.0)	Recessive	1.000	0.895	0.225–3.573			G/G	2 (2.4)	1 (6.7)	Recessive	0.399	0.350	0.030–4.124	
*TLR2*	rs56346547	MAF	0.268	0.133	Allele	0.166	2.383	0.787–7.215		rs5743794	MAF	0.372	0.433	Allele	0.525	0.774	0.352–1.704	
	A>C	A/A	42 (51.2)	11 (73.3)						G>A	G/G	33 (40.2)	3 (20.0)					
		A/C	36 (43.9)	4 (26.7)	Dominant	0.160	2.619	0.770–8.905			G/A	37 (45.1)	11 (73.3)	Dominant	0.159	0.371	0.097–1.418	
		C/C	4 (4.9)	0 (N/A)	Recessive	1.000	0.563	0.029–11.000			A/A	12 (14.6)	1 (6.7)	Recessive	0.685	2.400	0.288–19.960	
	rs62323831	MAF	0.311	0.467	Allele	0.097	0.516	0.234–1.136		rs3775073	MAF	0.305	0.367	Allele	0.503	1.320	0.585–2.978	
	G>A	G/G	36 (43.9)	3 (20.0)						A>G	A/A	40 (48.8)	4 (26.7)					
		G/A	41 (50.0)	10 (66.7)	Dominant	0.095	0.319	0.084–1.218			A/G	34 (41.5)	11 (73.3)	Dominant	0.160	0.382	0.112–1.298	
		A/A	5 (6.1)	2 (13.3)	Recessive	0.295	0.422	0.074–2.410			G/G	8 (9.8)	0 (N/A)	Recessive	0.351	3.537	0.194–64.600	
	rs13105517	MAF	0.354	0.400	Allele	0.627	0.821	0.370–1.822		rs2381289	MAF	0.445	0.467	Allele	0.827	0.917	0.420–2.001	
	G>A	G/G	31 (37.8)	4 (26.7)						T>C	T/T	23 (28.0)	4 (26.7)					
		G/A	44 (53.7)	10 (66.7)	Dominant	0.562	0.598	0.175–2.043			T/C	45 (54.9)	8 (53.3)	Dominant	1.000	0.933	0.269–3.229	
		A/A	7 (8.5)	1 (6.7)	Recessive	1.000	1.307	0.149–11.468			C/C	14 (17.1)	3 (20.0)	Recessive	0.723	0.824	0.205–3.306	
	rs3804100	MAF	0.366	0.433	Allele	0.483	0.754	0.343–1.661	*TLR10*	rs28393318	MAF	0.463	0.467	Allele	0.974	1.013	0.464–2.210	
	T>C	T/T	34 (41.5)	3 (20.0)						A>G	A/A	22 (26.8)	6 (40.0)					
		T/C	36 (43.9)	11 (73.3)	Dominant	0.153	0.353	0.092–1.347			A/G	44 (53.7)	4 (26.7)	Dominant	0.301	1.818	0.580–5.701	
		C/C	12 (14.6)	1 (6.7)	Recessive	0.685	2.400	0.288–19.960			G/G	16 (19.5)	5 (33.3)	Recessive	0.305	0.485	0.145–1.617	
*TLR4*	rs1927911	MAF	0.378	0.367	Allele	0.906	1.050	0.469–2.353		rs79030744	MAF	0.293	0.200	Allele	0.298	1.655	0.636–4.305	
	C>T	C/C	34 (41.5)	7 (46.7)						T>C	T/T	41 (50.0)	9 (60.0)					
		C/T	34 (41.5)	5 (33.3)	Dominant	0.708	1.235	0.409–3.731			T/C	34 (41.5)	6 (40.0)	Dominant	0.476	1.500	0.489–4.598	
		T/T	14 (17.1)	3 (20.0)	Recessive	0.723	0.824	0.205–3.306			C/C	7 (8.5)	0 (N/A)	Recessive	0.591	3.079	0.167–56.820	
	rs1927907	MAF	0.409	0.300	Allele	0.263	1.612	0.695–3.736		rs7658893	MAF	0.482	0.467	Allele	0.880	1.062	0.487–2.317	
	G>A	G/G	27 (32.9)	6 (40.0%)						A>G	A/A	21 (25.6)	5 (33.3)					
		G/A	43 (52.4)	9 (60.0%)	Dominant	0.595	1.358	0.438–4.209			A/G	43 (52.4)	6 (40.0)	Dominant	0.538	1.452	0.445–4.739	
		A/A	12 (14.6)	0 (N/A)	Recessive	0.203	5.496	0.308–97.950			G/G	18 (22.0)	4 (26.7)	Recessive	0.740	0.773	0.220–2.722	
	rs11536889	MAF	0.293	0.300	Allele	0.936	0.966	0.413–2.259		rs4274855	MAF	0.348	0.233	Allele	0.221	1.750	0.708–4.327	
	G>C	G/G	40 (48.8)	7 (46.7)						C>T	C/C	33 (40.2)	8 (53.3)					
		G/C	36 (43.9)	7 (46.7)	Dominant	0.880	0.919	0.305–2.769			C/T	41 (50.0)	7 (46.7)	Dominant	0.345	1.697	0.561–5.131	
		C/C	6 (7.3)	1 (6.7)	Recessive	1.000	1.105	0.123–9.901			T/T	8 (9.8)	0 (N/A)	Recessive	0.351	3.537	0.194–64.600	
*TLR9*	rs352140	MAF	0.470	0.367	Allele	0.298	1.529	0.685–3.414		rs10856838	MAF	0.201	0.167	Allele	0.805	1.260	0.448–3.540	
	C>T	C/C	21 (25.6)	5 (33.3)						T>A	T/T	51 (62.2)	10 (66.7)					
		C/T	45 (54.9)	9 (60.0%)	Dominant	0.538	1.452	0.445–4.739			T/A	29 (35.4)	5 (33.3)	Dominant	1.000	1.216	0.380–3.888	
		T/T	16 (19.5)	1 (6.7)	Recessive	0.458	3.394	0.415–27.778			A/A	2 (2.4)	0 (N/A)	Recessive	1.000	0.963	0.044–21.060	
										rs11096956	MAF	0.372	0.433	Allele	0.525	0.774	0.352–1.704	
										G>A	G/G	33 (40.2)	3 (20.0)					
											G/A	37 (45.1)	11 (73.3)	Dominant	0.159	0.371	0.097–1.418	
											A/A	12 (14.6)	1 (6.7)	Recessive	0.685	2.400	0.288–19.960	

* Allele: allele model; dominant: the minor allele dominant model; recessive: the minor allele recessive model. Abbreviations: IFX, infliximab; CD, Crohn’s disease; SNP, single nucleotide polymorphism; OR, odds ratio; CI, confidence interval; MAF, minor allele frequency; N/A, not applicable.

## Data Availability

The datasets generated during the current study are not publicly available because data sharing was not included in the informed consent form.
